# Assessment of Zooplankton Community Composition along a Depth Profile in the Central Red Sea

**DOI:** 10.1371/journal.pone.0133487

**Published:** 2015-07-17

**Authors:** John K. Pearman, Xabier Irigoien

**Affiliations:** Red Sea Research Center, KAUST- King Abdullah University of Science and Technology, Thuwal, Saudi Arabia; The Evergreen State College, UNITED STATES

## Abstract

The composition of zooplankton in the water column has received limited attention in the main body of the Red Sea and this study investigates the change in the community both spatially and temporally across 11 stations in the central Red Sea. Using molecular methods to target the v9 region of the 18S rRNA gene a total of approximately 11.5 million reads were sequenced resulting in 2528 operational taxonomic units (OTUs) at 97% similarity. The phylum Arthropoda dominated in terms of reads accounting for on average 86.2% and 65.3% for neuston nets and vertical multinets respectively. A reduction in the number of OTUs was noticed with depth for both total metazoa and Maxillopoda whilst there was also a significant change in the composition of the Maxillopoda community. The genus *Corycaeus* had a higher proportion of reads in the epipelagic zone with *Pleuromamma* becoming increasingly dominant with depth. No significant difference was observed in the community between night and day sampling however there was a significant difference in the zooplankton community between two sampling periods separated by 10 days.

## Introduction

Zooplankton play a substantial role in marine food webs and biogeochemical pathways [1]. Alterations in the planktonic community composition have been shown to have impacts higher up the trophic chain [2]. However whole community studies of zooplankton are limited and this is especially true of the Red Sea, a semi confined sea that combines high temperature, salinity and oligotrophic conditions [3]. The majority of studies undertaken on zooplankton in the Red Sea have concentrated on its northern extent and especially the Gulf of Aqaba [4, 5, 6]. These have included reports on zooplankton distributions throughout the water column [6, 7] as well as those at the surface [8]. However, studies in the central and southern reaches of the Red Sea are lacking. Böttger Schnack [9, 10] studied micro and mesozooplankton focusing on non-calanoid copepods whereas Schneider et al [11] described the abundance and size structure of the community. Whilst Pearman et al [12] studied zooplankton communities associated with coral reefs. The complex taxonomy of tropical communities and limited number of studies has meant that the overall diversity of the zooplankton assemblage has been poorly described.

Molecular techniques have enabled biodiversity studies to be undertaken at a greater depth and scale. Environmental DNA sampling relies on the extraction of bulk DNA and amplicon sequencing of a common molecular maker, such as the small subunit ribosomal RNA gene (SSU rRNA) or mitochondrial cytochrome oxidase I (COI). Molecular techniques were first utilized to study microbial populations (e.g. [13, 14]), but have more recently been applied to marine metazoan communities (for example [12, 15, 16, 17]. It is a technique, which allows analysis of organisms across a broad taxonomic range based on sequence similarities to reference databases. Molecular techniques have the possibility to simplify the analysis of marine zooplankton communities by allowing the whole community to be assessed simultaneously without the need for specific experts in morphological taxonomy of each taxa. As well as this the identification of larval and juvenile stages, which are complicated to differentiate morphologically would be able to be identified using genetic methods.

In this study, using high throughput sequencing methods we investigate the diversity of zooplankton communities in the central Red Sea region. To target the whole of the zooplankton community we used general eukaryotic primers targeting the v9 hypervariable region of the 18S rRNA gene [18]. Clustering of Operational Taxonomic Units (OTUs) was undertaken at 97% similarity so that OTU richness was most closely aligned to species richness [16, 19]. To date there have been few studies that have investigated the whole zooplankton community in the main body of the Red Sea and therefore this study provides a basic understanding of the community structure and improves our knowledge of the marine ecosystem in the Red Sea.

## Methods

### Sampling

**Fig 1:**
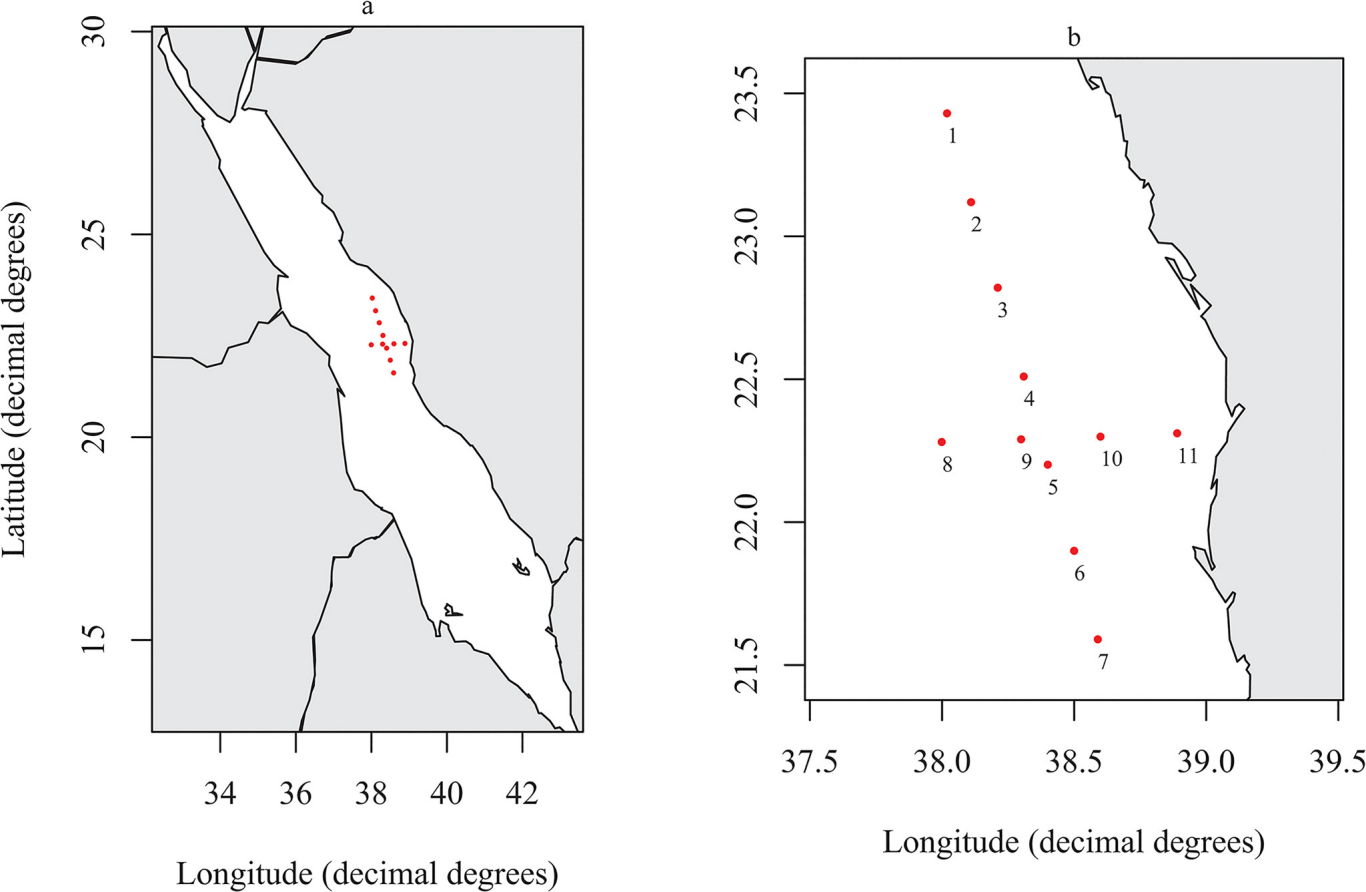
Sampling stations. Positions of sampling stations showing a) Positions relative to the Red Sea and b) the position of individual stations. Figure plotted in R using the package *maps* [20].

Samples were collected during March—April 2013 in the central Red Sea region (S1 Table; Fig 1) during the KAUST Red Sea Research Cruise 2013 aboard the RV Aegaeo. Permits for sampling in Saudi Arabian waters were obtained from the Saudi Arabian coastguard. No specific permissions were required, as the study did not involve endangered or protected species. A total of 11 stations were sampled in the region with the stations being repeated 10 days after the first sampling. Zooplankton samples were collected using two methods. Firstly depth profiles were obtained using a multinet system (Hydro-Bios, Kiel) fitted with a mesh size of 64 μm and a CTD profiler attached. Vertical hauls were carried out between 500 m and the surface with a towing speed of 1 m s^-1^. The depths at which nets were triggered are detailed in S1 Table. Secondly, surface zooplankton samples were obtained using a neuston net with a 64 μm mesh size towed at approximately 3 knots for 10 min. Subsequent to sampling the samples were stored at 4°C in absolute ethanol.

### DNA extraction and sequencing

DNA was extracted based on the methods described in [21]. Ethanol was removed from the samples prior to the addition of ATL lysis buffer (Qiagen). The whole sample was transferred to a pestle and mortar and crushed until the biomass was liquidized. To complete the lysis of the sample the resulting cell suspension was incubated overnight at 55°C with 20 μl proteinase K (20 mg/mL). For samples with larger biomass the addition of proteinase K was scaled up. Phenol:chloroform:iso amyl alcohol (IAA) was added in equal volume to the sample, mixed and centrifuged for 5 min. The aqueous layer was removed and a second round of phenol:chloroform:IAA extraction was undertaken followed by a round of chloroform:IAA. The aqueous layer was removed and DNA precipitated using 2.5 volumes ethanol and 0.1 volumes sodium acetate at – 20°C. DNA was washed in 70% ethanol and resuspended in autoclaved MilliQ water.

Amplification of eukaryotic DNA was achieved using the general eukaryotic primers designed by [18]. The forward primer 1389F was combined with the reverse primer 1510R. All primers had an Iontorrent tag (instead of a Roche 454 tag as used by [18]) and a 10 bp barcode. PCRs were undertaken in duplicate in 50 μl reaction volumes containing 2.5U Taq polymerase (Invitrogen), 1XTaq reaction Buffer, 200μM dNTPs (Invitrogen), 1.5mM MgSO_4_, 0.05 mg Bovine Serum Albumin (BSA) and 0.2μM of each primer. PCR conditions were adapted from [18]. An initial denaturation step of 3 min at 94°C was followed by 30 cycles of 94°C for 30 sec, 57°C for 45 sec and 72°C for 45 sec. This is followed by a final extension at 72°C for 10 min. A no template negative control was also run. Duplicate PCR reactions were combined and the PCR band visualized on a 1% agarose gel before being excised and purified with Qiagen’s gel extraction kit. Purified samples were pooled, having been equalized to 50ng (measured using a dsDNA high sensitivity kit for a Qubit 2.0 Fluorometer (Life Technologies)), according to barcode. A maximum of 15 unique barcodes were pooled together and multiplexed samples were analyzed for purity using an Agilent 2100 bioanalyzer machine. Emulsion PCR for IonTorrent sequencing was undertaken, using an Ion PGM Template OT2 400 Kit on a Ion One Touch system 2.0 following manufacturers protocols, with 15 pM of template amplicons. After enrichment in a Ion one touch ES system the templates were loaded on a 318 V2 chip and sequenced at the King Abdullah University of Science and Technology (KAUST), Thuwal, Saudi Arabia, core facility on an IonTorrent PGM machine using an Ion PGM Sequencing 400 Kit. Raw sequence reads were deposited in the NCBI Sequence Read Archive under the experiment accession number: SRP056532.

### Sequence analysis and bioinformatics

Raw sequences were automatically demultiplexed, based on barcode, on the IonTorrent PGM machine and each sample was filtered individually using the QIIME (version 1.8) software package [22]. Parameters for quality filtering were minlength = 100, maxlength = 200, quality = 25, windowsize = 25, max homopolymers = 6 and max forward primer mismatch = 1, barcode = 0. Filtered sequences were concatenated and clustering into OTUs occurred through a two-step process. Firstly, using the trie function of the QIIME version of the CD-HIT [23] algorithm, sequences were preclusted and the longest representative sequence of each precluster was selected. Secondly de novo clustering of these representative sequences using the USEARCH (version 5.2.236) [24] algorithm at 97% identity was undertaken where clusters had to include at least 2 sequences. Representative sequences (longest) were selected for the USEARCH OTUs and checked for chimeras against a reference database (SILVA 119 [25] using UCHIME [26]). Taxonomic assignments were made in QIIME against the SILVA 119 database using the naïve Bayesian classifier rdp [27]. To assess the composition across different stations reads were rarefied multiple times (n = 100) at an even depth (20,000 reads) and the average taken. Diversity statistics were undertaken using QIIME and the R (v 3.0.2 (R development core team)) packages *vegan* [28] and *phyloseq* [29]. Weighted and unweighted UniFrac [30] distance matrices were constructed in *phyloseq* based on the QIIME OTU table and phylogenetic tree. Non-metric multidimensional scaling (NMDS) plots were constructed with *phyloseq* and statistical analysis of the distance matrices was calculated using PERMANOVA (adonis in *vegan*).

## Results

**Fig 2:**
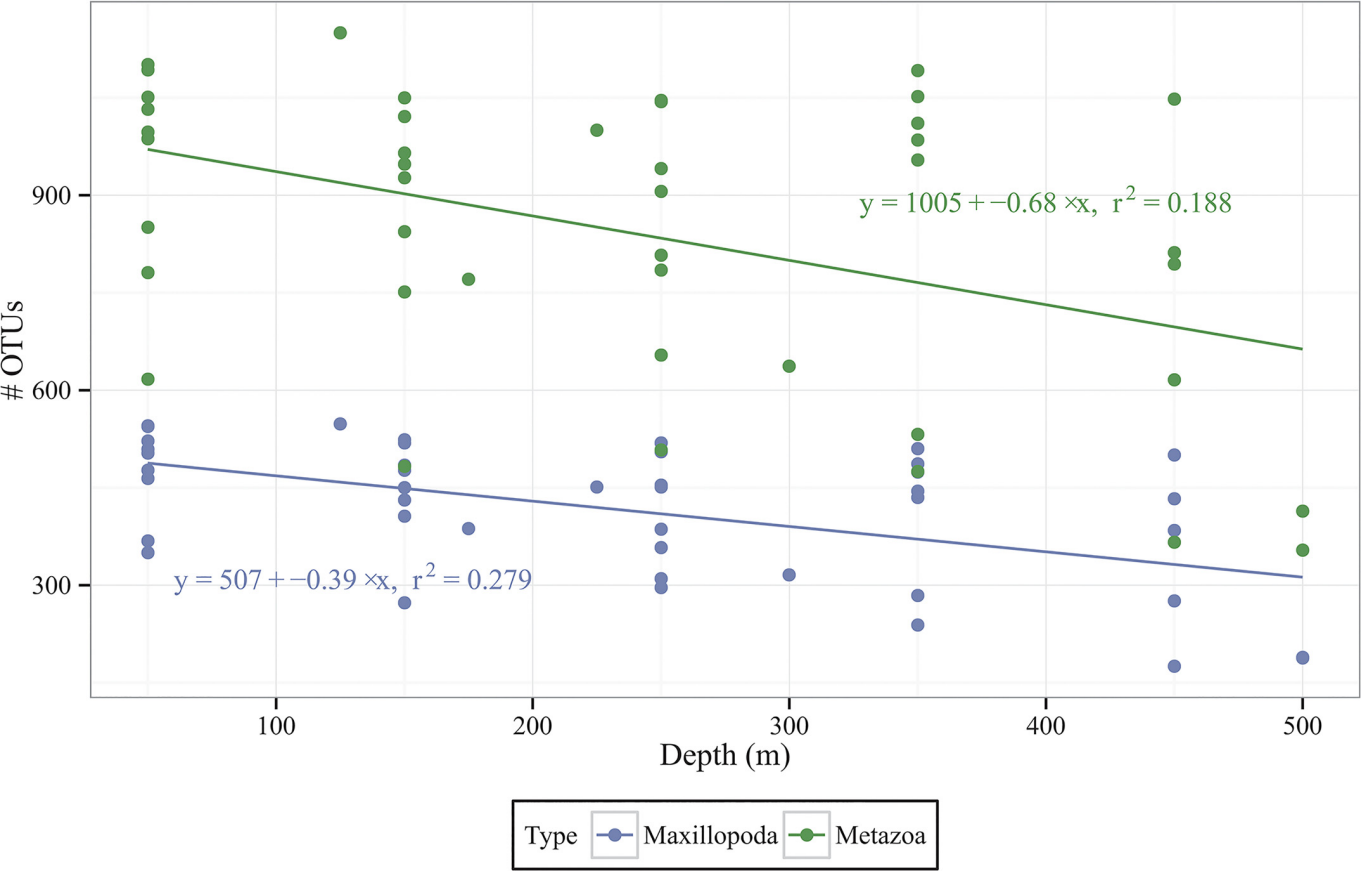
Association of OTU counts with depth. Number of OTUs for both Metazoa and Maxillopoda against depth. The average depth at which each multinet was open was used.

A total of 11,693,628 reads were obtained across all the samples and clustering resulted in a total of 2528 metazoan OTUs at 97% similarity, subsequent to the removal of 110 OTUs which were designated as chimeras as well as those which were not taxonomically classified as metazoa. In order for samples to be compared the metazoan reads were rarefied multiple times (n = 100) at an even depth (20,000 reads). After rarefaction a total of 2079 OTUs were observed. The majority of these OTUs were grouped in the phylum Arthropoda (1285 OTUs; 62% of total OTUs), and particularly the class Maxillopoda (886 OTUs; 43% of total OTUs). Other phyla accounting for more than 100 OTUs include the Cnidaria (131 OTUs; 6.3% of total OTUs), Urochordata (118 OTUs; 5.7% of total OTUs) and Mollusca (104 OTUs; 5.0% of total OTUs). Comparing alpha diversity down the depth profile it was found that there was a significant decrease in the numbers of both metazoan (F = 9.485; p = 0.004) and Maxillopoda (F = 15.9; p < 0.001) OTUs with depth (Fig 2).

**Fig 3:**
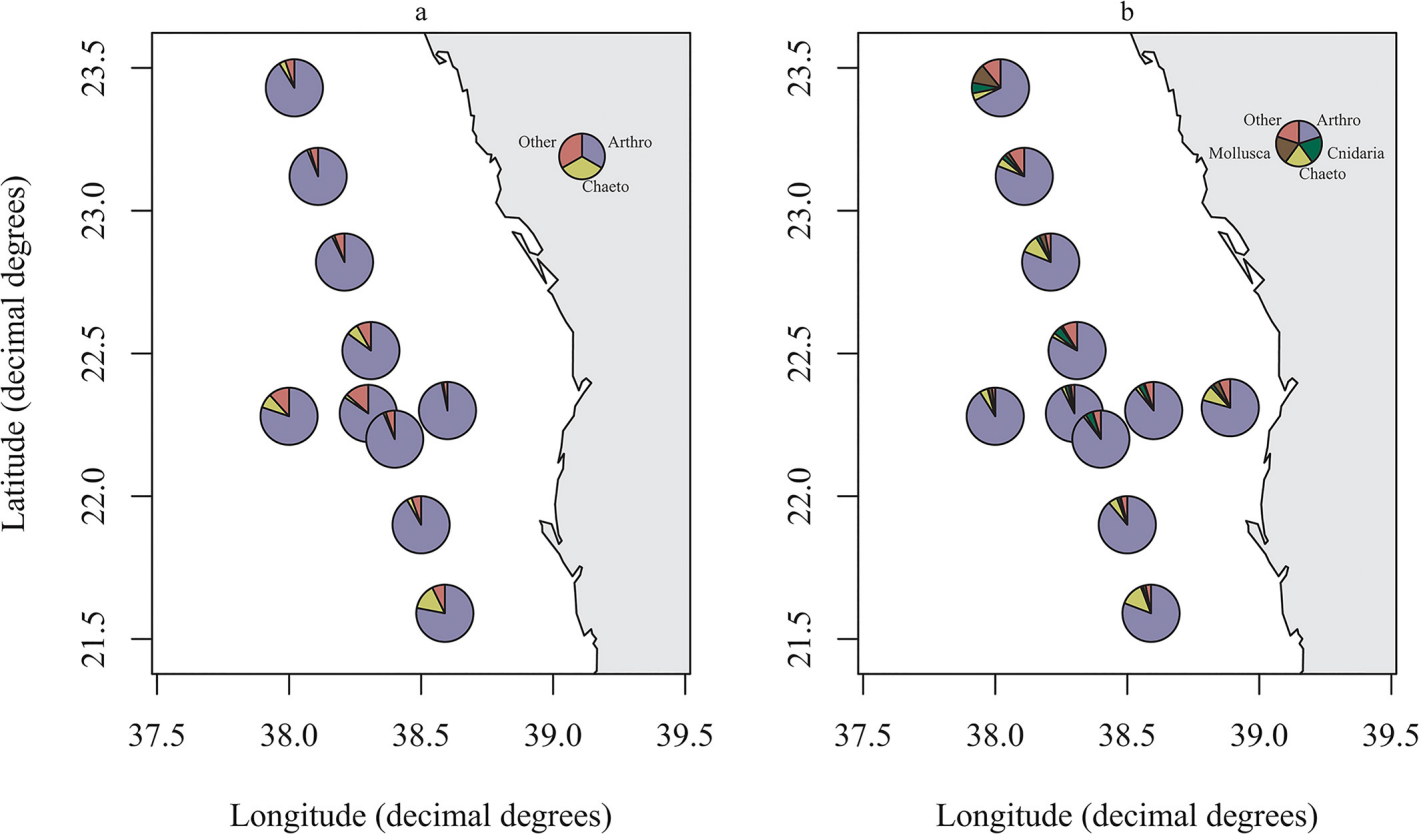
Zooplankton composition for a) neuston and b) neuston repeat. Composition of zooplankton at the phyla level for a) the neuston surface samples and b) the neuston surface repeated samples. Arthro = Arthropoda; Chaeto = Chaetognatha. Other accounts for those phyla, which accounted for less than 3% of a sample or OTUs that were not assigned to the phyla level. For station designations refer to S1 Table. Figure plotted in R using the package *maps* [20].

At all stations, samples taken using the neuston net were dominated by the phyla Arthropoda (86.2%) (Fig 3A and 3B). Smaller proportions of reads were attributed to Chaetognatha (average 4.9%), Cnidaria (average 2.2%), Urochordata (average 1.7%) and Mollusca (average 1.6%). Mollusca and Cnidaria were more prevalent in the second sampling period (Fig 3B). On a finer taxonomic scale, the neuston nets were dominated by the class Maxillopoda (phyla Arthropoda) (average 76.9%), with smaller contributions to the samples from Malacostraca (phyla Arthropoda) (average 8.2%). During the second sampling period the Maxillopoda genus *Corycaeus* was more prevalent than in the first, accounting for an average of 10.23% and 3.24% of the community respectively.

**Fig 4:**
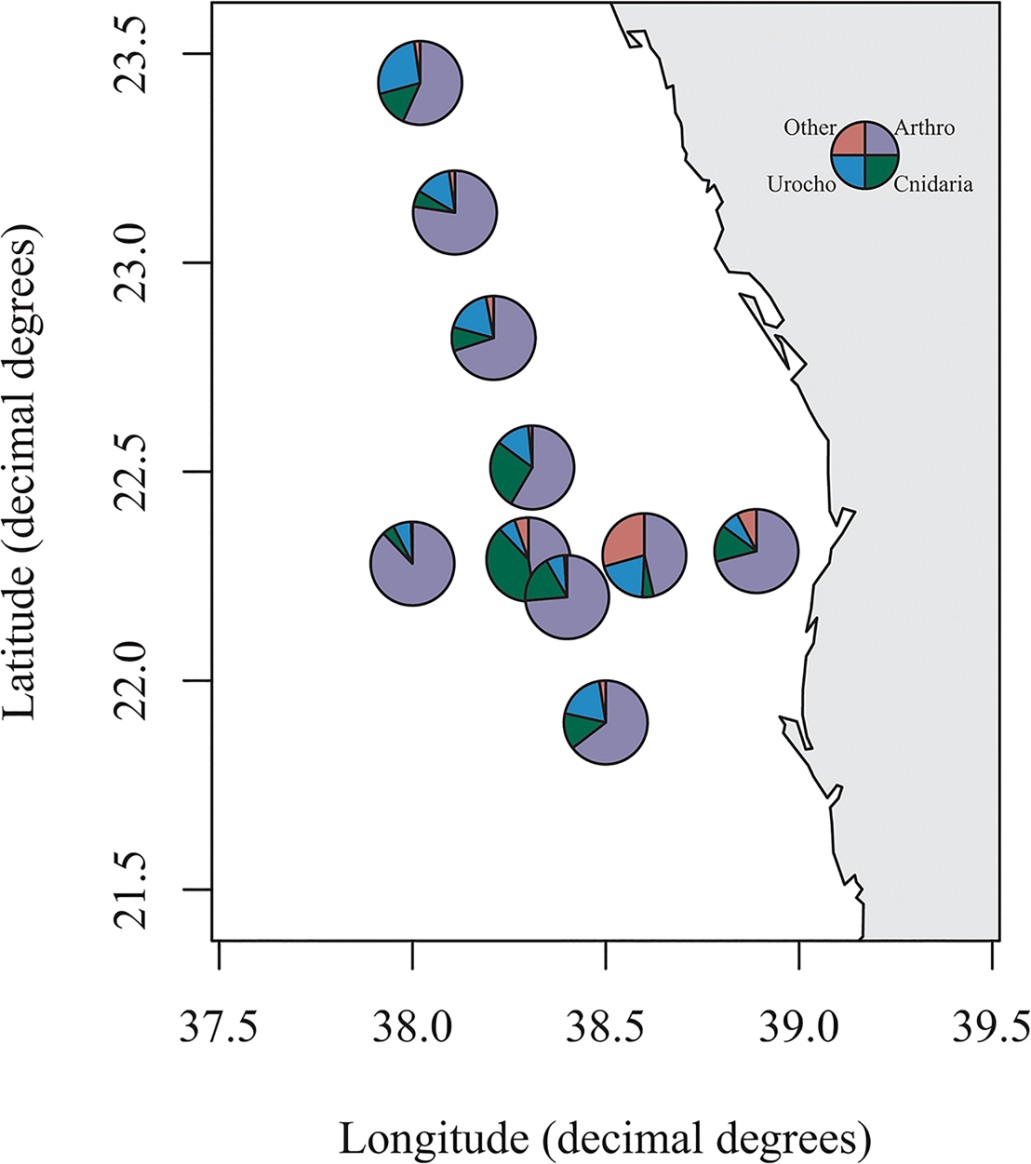
Metazoa composition in multinet samples. Combined composition of metazoa at each station in the multinet samples. Arthro = Arthropoda; Urocho = Urochordata. Other accounts for those phyla, which accounted for less than 3% of a sample or OTUs, which were not classified at the phyla level. For station designations refer to S1 Table. Figure plotted in R using the package *maps* [20].

**Fig 5:**
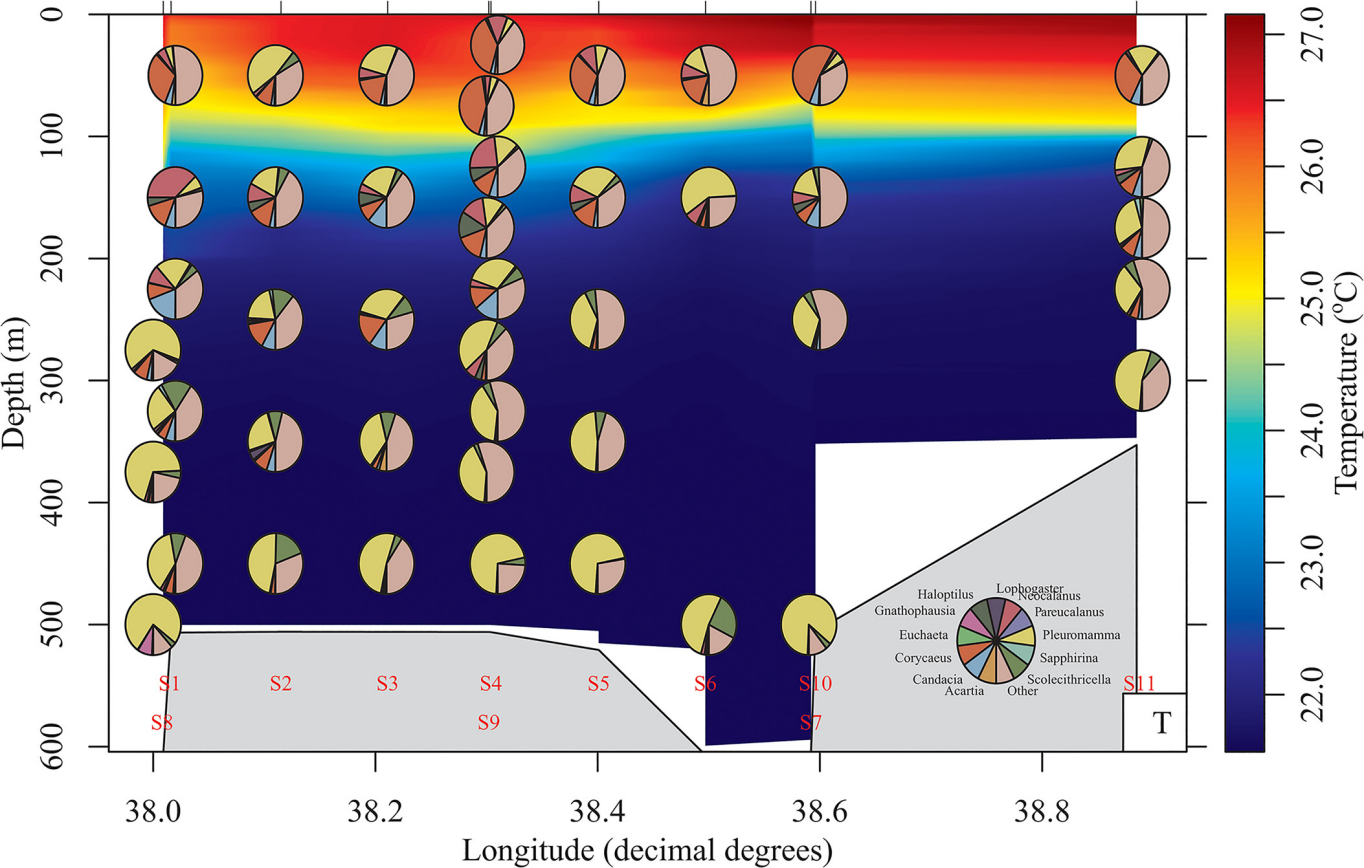
Depth profile of Maxillopoda taxa. Depth profile of the composition of the Maxillopoda genera in the multinet samples. Pies are plotted at the average depth for which the net was open except where stations are close in longitude were one station is plotted at the 1st quartile whilst the other is plotted at the 3rd quartile to avoid overlap. Background is temperature (°C). Others includes those genera with < 1% reads in at least one sample and those OTUs classified as Maxillopoda but which could not be assigned to genus level. Figure plotted with R package *oce* [31].

On average throughout the water column Arthropoda were the dominant component of the plankton averaging 65.3%, however Cnidaria also accounted for a substantial proportion (average 15.1%) whilst Urochordata also accounted for a sizeable proportion of reads in several stations (Fig 4). Table 1 shows the taxonomic designations (at genus level where possible) of the most dominant OTU for the metazoan phyla down the water column. Maxillopoda were the dominant class of Arthropoda throughout the depth profile, however the genera present differed through the water column. A higher proportion of reads were attributed to the genus *Pleuromamma* at deeper depths than at shallower ones whilst the genus *Corycaeus* was more prominent in the top 100m (Fig 5).

**Table 1:** Genus assignments for the dominant OTUs. Taxonomic assignation of the dominant genus (where identified) for the metazoa phyla at each sampling point. See S1 Table and Fig 1 for station designations. Blank designations are where the dominant OTU could not be assigned to the genus level.

**Net**	Annelida	Arthropoda	Brachiopoda	Bryozoa	Cephalochordata	Chaetognatha	Cnidaria	Ctenophora	Echinodermata	Hemichordata	Mollusca	Platyhelminthes	Urochordata
**M1.1**	* *	*Nyctiphanes*	* *	* *	* *	*Decipisagitta*	* *	*Mertensia*	* *	* *	* *	* *	*Oikopleura*
**M1.2**	*Tomopteris*	*Conchoecia*	* *	*Smittoidea*	* *	*Decipisagitta*	* *	* *	*Ophiocoma*	* *	*Ptychobela*	*Stylochus*	* *
**M1.3**	* *	* *	* *	*Smittoidea*	*Branchiostoma*	*Decipisagitta*	* *	* *	* *	* *	* *	*Stylochus*	* *
**M1.4**	* *	*Neocalanus*	* *	*Smittoidea*	* *	*Aidanosagitta*	* *	* *	* *	* *	* *	* *	* *
**M1.5**	* *	* *	*Chloeia*	*Smittoidea*	* *	*Aidanosagitta*	* *	* *	* *	* *	* *	* *	* *
**M2.1**	*Tomopteris*	*Pleuromamma*	* *	*Smittoidea*	* *	*Decipisagitta*	* *	*Mertensia*	* *	* *	* *	* *	* *
**M2.2**	* *	*Conchoecia*	* *	* *	* *	*Decipisagitta*	* *	*Mertensia*	* *	* *	* *	* *	*Ritteriella*
**M2.3**	* *	* *	*Chloeia*	* *	* *	*Decipisagitta*	* *	*Hormiphora*	* *	* *	* *	* *	* *
**M2.4**	*Tomopteris*	* *	*Chloeia*	* *	* *	*Decipisagitta*	* *	* *	*Lytechinus*	* *	* *	* *	* *
**M2.5**	* *	*Pleuromamma*	* *	*Smittoidea*	* *	* *	* *	*Mertensia*	* *	* *	*Creseis*	* *	*Soestia*
**M3.1**	*Tomopteris*	*Pleuromamma*	* *	* *	* *	*Decipisagitta*	* *	*Hormiphora*	*Fellaster*	*Glandiceps*	* *	* *	* *
**M3.2**	* *	* *	* *	* *	* *	*Decipisagitta*	* *	*Hormiphora*	* *	*Glandiceps*	* *	* *	* *
**M3.3**	*Tomopteris*	*Conchoecia*	* *	* *	*Branchiostoma*	*Decipisagitta*	* *	*Hormiphora*	* *	* *	* *	* *	* *
**M3.4**	*Tomopteris*	* *	*Chloeia*	* *	* *	*Decipisagitta*	* *	* *	* *	*Glandiceps*	* *	* *	*Oikopleura*
**M3.5**	* *	* *	* *	* *	* *	*Aidanosagitta*	* *	* *	*Eucidaris*	*Glandiceps*	* *	*Stylochus*	* *
**M4.1**	* *	*Pleuromamma*	* *	* *	* *	*Aidanosagitta*	* *	* *	* *	* *	* *	* *	*Ritteriella*
**M4.2**	* *	* *	* *	*Smittoidea*	* *	*Decipisagitta*	* *	* *	* *	* *	* *	* *	*Ritteriella*
**M4.3**	* *	*Conchoecia*	* *	*Smittoidea*	* *	*Decipisagitta*	* *	*Mertensia*	* *	* *	* *	* *	* *
**M4.4**	* *	* *	* *	*Smittoidea*	* *	*Aidanosagitta*	* *	* *	* *	* *	*Ptychobela*	* *	* *
**M4.5**	* *	* *	*Chloeia*	* *	* *	*Aidanosagitta*	* *	* *	* *	* *	*Creseis*	* *	* *
**M5.1**	* *	*Pleuromamma*	* *	* *	* *	*Decipisagitta*	*Aegina*	*Hormiphora*	* *	* *	* *	*Prostheceraeus*	*Oikopleura*
**M5.2**	* *	*Pleuromamma*	* *	* *	* *	*Decipisagitta*	* *	*Mertensia*	* *	* *	* *	* *	*Soestia*
**M5.3**	* *	* *	* *	*Smittoidea*	* *	*Decipisagitta*	* *	*Mertensia*	* *	* *	* *	*Prostheceraeus*	* *
**M5.4**	*Typhloscolex*	*Conchoecia*	* *	*Smittoidea*	* *	*Decipisagitta*	* *	* *	* *	* *	* *	* *	* *
**M5.5**	* *	* *	* *	*Smittoidea*	* *	*Aidanosagitta*	* *	* *	*Ophiocoma*	* *	* *	*Stylochus*	* *
**M6.1**	* *	*Conchoecia*	* *	* *	* *	*Decipisagitta*	* *	*Mertensia*	* *	* *	*Creseis*	* *	*Oikopleura*
**M6.4**	* *	*Pleuromamma*	* *	* *	* *	*Aidanosagitta*	* *	* *	* *	* *	*Desmopterus*	*Stylochus*	*Oikopleura*
**M6.5**	*Tomopteris*	* *	* *	*Smittoidea*	* *	*Aidanosagitta*	* *	* *	* *	* *	* *	*Stylochus*	*Oikopleura*
**M7.1**	* *	*Pleuromamma*	* *	* *	* *	*Decipisagitta*	* *	*Mertensia*	* *	* *	* *	* *	* *
**M8.1**	*Tomopteris*	*Pleuromamma*	* *	* *	* *	*Decipisagitta*	* *	*Mertensia*	* *	* *	*Creseis*	* *	*Soestia*
**M8.2**	*Tomopteris*	*Pleuromamma*	* *	* *	* *	*Decipisagitta*	* *	*Mertensia*	* *	* *	* *	* *	*Oikopleura*
**M8.3**	*Tomopteris*	*Pleuromamma*	* *	* *	* *	*Decipisagitta*	* *	*Mertensia*	* *	* *	*Desmopterus*	*Prosogonotrema*	* *
**M9.2**	* *	* *	* *	* *	* *	*Decipisagitta*	*Aegina*	*Mertensia*	* *	* *	*Desmopterus*	* *	*Ritteriella*
**M9.3**	*Tomopteris*	*Conchoecia*	* *	* *	* *	*Decipisagitta*	* *	*Hormiphora*	* *	* *	* *	* *	* *
**M9.4**	* *	* *	* *	* *	* *	*Decipisagitta*	* *	* *	* *	* *	* *	*Stylochus*	* *
**M9.5**	* *	*Corycaeus*	*Chloeia*	*Smittoidea*	* *	*Aidanosagitta*	* *	* *	* *	* *	* *	* *	* *
**M10.3**	*Tomopteris*	* *	* *	* *	* *	*Decipisagitta*	* *	*Mertensia*	* *	* *	*Desmopterus*	*Prostheceraeus*	* *
**M10.4**	*Tomopteris*	*Conchoecia*	* *	*Smittoidea*	* *	*Aidanosagitta*	* *	* *	* *	* *	* *	*Stylochus*	*Ritteriella*
**M10.5**	* *	*Corycaeus*	*Chloeia*	* *	* *	*Aidanosagitta*	* *	* *	* *	* *	* *	*Stylochus*	* *
**M11.1**	* *	*Pleuromamma*	* *	* *	* *	*Decipisagitta*	* *	*Mertensia*	* *	* *	* *	* *	*Oikopleura*
**M11.2**	* *	* *	* *	* *	* *	*Decipisagitta*	* *	* *	* *	*Glandiceps*	*Sinezona*	* *	*Oikopleura*
**M11.3**	*Tomopteris*	* *	* *	* *	* *	* *	* *	* *	* *	* *	* *	* *	* *
**M11.4**	*Tomopteris*	* *	*Chloeia*	* *	* *	* *	* *	* *	* *	* *	*Ostrea*	* *	* *
**M11.5**	* *	* *	* *	*Smittoidea*	* *	*Aidanosagitta*	* *	* *	* *	* *	* *	* *	* *

**Fig 6:**
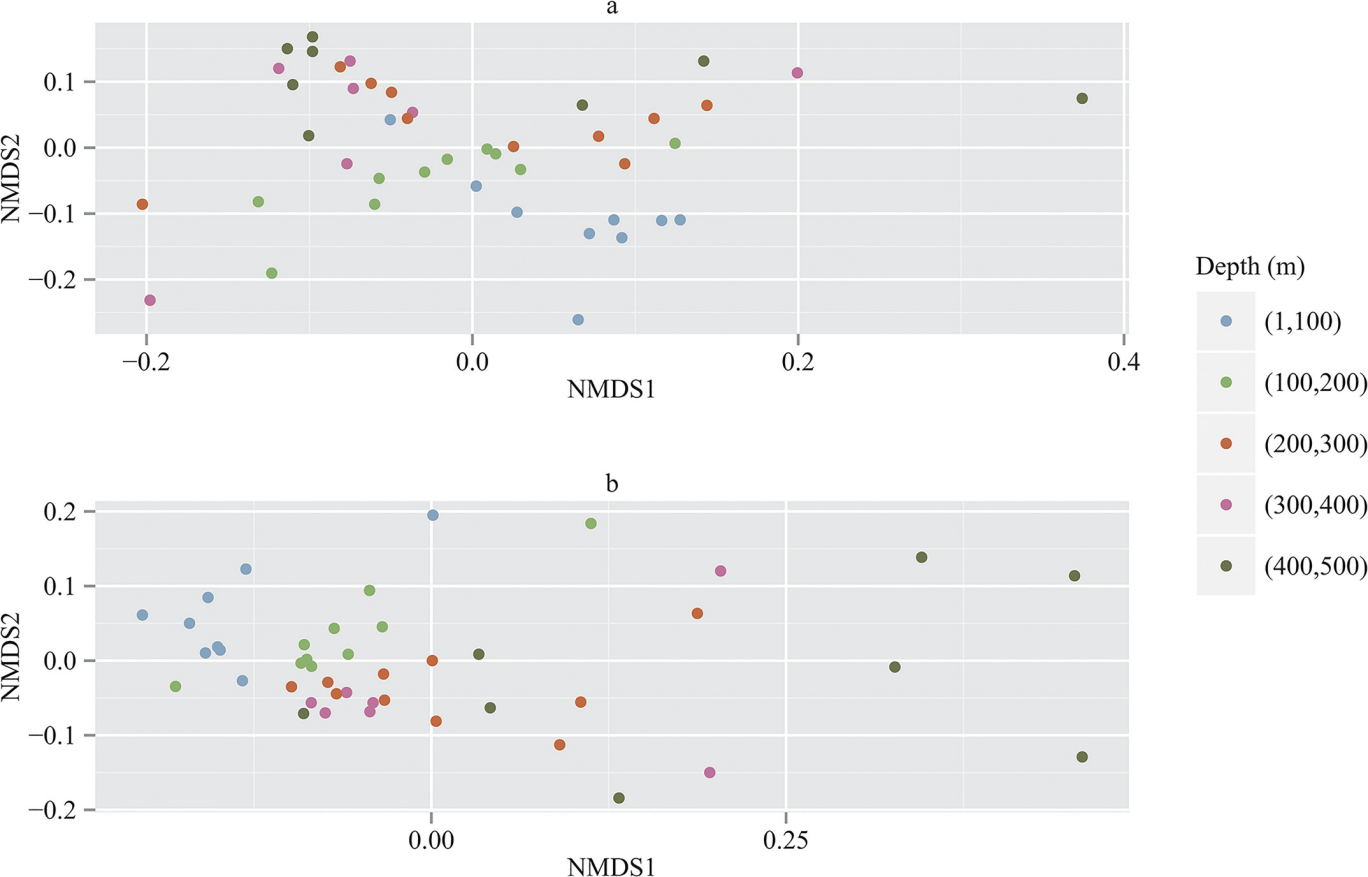
NMDS plot for a) weighted and b) unweighted UniFrac for multinet samples. NMDS plot based on a) weighted and b) unweighted UniFrac distance matrices showing clustering by depth. Samples were categorized based on the average depth, which the net was opened.

To assess statistical differences in the zooplankton community samples, those, which had a full depth profile (3 night stations and 3 day stations), were analyzed. Adonis analysis was undertaken to investigate the effects of depth and time of day. Both weighted and unweighted UniFrac results showed that there was a significant difference with depth (weighted: F = 3.17, p < 0.001; unweighted F = 2.588, p < 0.001) with Fig 6A + 6B showing a NMDS of all sampling points. There was no significant effect of time of day (weighted: F = 2.018, p = 0.057; unweighted F = 1.514, p = 0.058). A similar finding was found when just considering the dominant group Maxillopoda with depth being found to be significant (weighted: F = 12.353, p < 0.001; unweighted F = 3.084, p < 0.001) whilst time of day was not significant (weighted: F = 0.782, p = 0.428; unweighted F = 1.197, p = 0.262).

**Fig 7:**
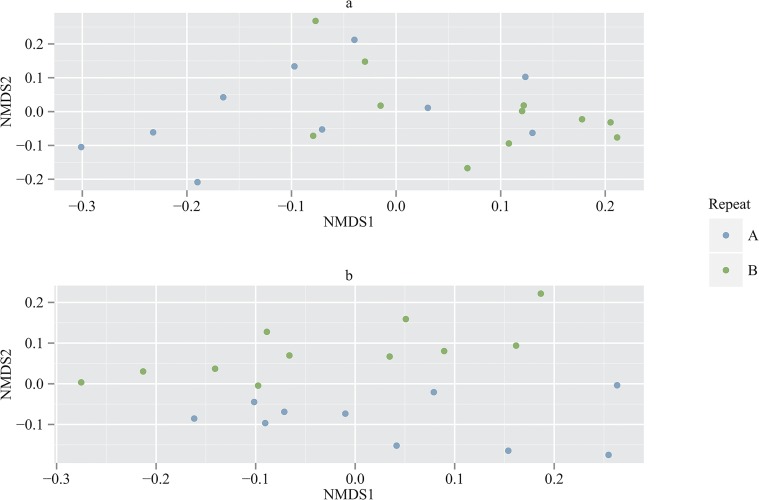
NMDS plot for a) weighted and b) unweighted UniFrac for neuston samples. NMDS plot based on a) weighted and b) unweighted UniFrac distance matrices showing clustering of the sampling period. A is the sampling period between 25^th^ March 2013 and 27^th^ March 2013 whilst B is the sampling period between 3^rd^ April 2013 and 5^th^ April 2013.

In the neuston nets there was also found to be no significant difference in the metazoan community depending on the time of day of sampling (after 9 samples were randomly chosen for both night and day) (weighted: F = 0.789, p = 0.579; unweighted: F = 0.886, p = 0.612). However a statistical difference was observed between the two sampling periods (weighted: F = 2.738, p = 0.014; unweighted: F = 2.289, p = 0.004) between the first 10 stations (Station 11 only had results from the repeat sampling period) (Fig 7A + 7B). No significant trends (p > 0.05) were observed between distance and similarity of the zooplankton community for either UniFrac distance measure or sampling period.

## Discussion

The present study uses high throughput sequencing technology to provide basic information on the vertical profile of zooplankton in the central Red Sea. Morphological investigations often focus on the abundance of each species in the zooplankton community. However using next generation sequencing techniques the correlation between reads and abundance cannot be presumed. Previous genetic studies of the zooplankton community targeting the 18S rRNA gene showed that for bivalve and decapod larvae metagenetic reads correlated more with morphological biomass than abundance [16], whilst [15] found a correlation between copepod LSU reads and dry weight. Although this correlation could be affected by biases such as primer mismatches or gene copy numbers [32, 33] it can give an approximate representation of changes in the biomass of the zooplankton community. The dominant phyla in the current study were Arthropoda in both the neuston and vertical profiles with Cnidaria also accounting for a substantial proportion of reads (Figs 3 and 4). Urochordata also accounted for a large proportion of reads in the vertical nets with Chaetognatha and Mollusca observed in the neuston nets. Similar groups were found to be the most abundant in previous studies in the Red Sea (e.g. [4, 5, 34, 35]) and in other water bodies including the subtropical Pacific [36] and UK coastal waters [16]. Copepods were the dominant component of the Arthropoda in both the neuston and vertical net samples. This has been previously shown in a molecular study of zooplankton associated with coral reefs in the Red Sea [12], morphological studies undertaken in the Gulf of Aden and Red Sea (e.g. [6, 11, 35, 37]) and in other marine basins (e.g. Mediterranean: [38]; subtropical Pacific: [36]; Caribbean: [39]). The dominant genus identified in the epipelagic zone was the Poecilostomatoid copepod *Corycaeus*, which has also been found to be present in other Red Sea studies [37] although other genera such as *Clausocalanus* and *Ctenocalanus* [7, 40] are often more abundant. Lindeque [16] has previously reported that *Clauso-/Ctenocalanus* accounted for a higher proportion of morphologically identified samples compared with molecular techniques. The differences were attributed to high abundance relative to biomass of juveniles of these species and similar explanations could account for the low proportion of these genera in the current study with an increase in copepodit stages of *Clausocalanus* spp. having been shown in March/April in the Gulf of Aqaba [41]. The calanoid *Pleuromamma* was dominant in the mesopelagic zone, which is in agreement with previous studies [7, 40] (Fig 5).

In the current study, in agreement with previous studies [6] we found that there was no significant difference in both the metazoan and maxillopoda community between samples taken at night and those taken during the day. However the dominant Maxillopoda genus *Pleuromamma* was observed to have higher abundances of reads in the deeper samples, as has been observed previously in the Red Sea [40]. In agreement with the fact this genus undertakes diel vertical migrations [40, 42, 43] higher abundances were observed in the epipelagic zone of those stations where sampling occurred during the night. However, large proportions of reads were still observed in the mesopelagic zone suggesting either a proportion of the population was non-migratory as has been observed in various species of this genus [43] or different life cycle distribution patterns [44].

A significant decrease in the number of metazoan and Maxillopoda OTUs was noticed with depth (Fig 2), which is in agreement with previous investigations in subtropical regions [6, 40, 45, 46] but shows a different distribution from that observed for zooplankton in the Amundsen and Makarov Basins where the maximum was observed between 500–750m [47].

Whilst studies have shown changes in the abundance of zooplankton in the Red Sea on a seasonal scale [4, 5, 7, 37] we are able to show the dynamic nature of the planktonic community with statistically significant changes in the neuston community between sampling of the same stations 10 days apart. The difference in the community appeared to be due to the change in rare taxa with the unweighted UniFrac, which favors rare taxa being more significant than the weighted UniFrac. However, the genus *Corycaeus* accounted for 10.23% of the reads in the repeat sampling and only 3.24% in the first period of sampling. This suggests that studies incorporating short time periods between sampling efforts will be required to fully understand the changes in the plankton community in the Red Sea.

One advantage of molecular techniques over those based on morphology is that larval and juvenile specimens can be hard to identify morphologically [16] whilst there is no differentiation in life stage when using molecular techniques. El Sherbiny et al [5] found that adult copepods only constituted 22.3% of the total copepods meaning identification of a substantial proportion of the zooplankton community would be problematic. The focus on adult copepods is a fundamental issue in morphological studies and could mean that there are misrepresentations of zooplankton diversity with [16] suggesting that next generation sequencing technologies may reveal double the copepod diversity.

Whilst sequencing technologies have their advantages there are currently limitations for their use in ecological studies. For example, in the current study, on average 40% of the reads from the multinet samples classified as Maxillopoda could not be classified at a lower taxonomic level. Genera such as *Oncaea*, which have previously been shown to be a key component of the Red Sea zooplankton community [5], are absent from reference databases meaning that reads would fall into the unknown category. Lindeque et al [16] found that off the UK coast only half the possible local species of *Oithona* were present in the database and this limits the ability to classify species at low taxonomic levels and missing sequences of representative species from the Red Sea may partly account for the lower proportion of this genera observed in the current study despite high abundances previously reported [35]. This problem is likely to be exacerbated in regions, such as the Red Sea where the diversity of zooplankton has received considerably less attention.

In summary, this study has increased the amount of knowledge available on the diversity of zooplankton in the central Red Sea. Whilst there are still limitations in the use of molecular techniques to study the diversity of metazoans this study has shown that the techniques are able to discriminate changes in the structure of the zooplankton community not only in time but also down a depth profile at different taxonomic levels. In the future with the collaboration of molecular ecologists and morphological taxonomists the gaps in reference databases can be closed leading to an improvement in the speed and scale at which ecological studies can be undertaken.

## Supporting Information

S1 Table Ancillary data for neuston and multinet samples including temperature, conductivity, salinity, depth, latitude, longitude, day/night and date of sampling. (CSV)
